# Right ventricular outflow tract obstruction caused by right ventricular fibroma in a 5-month-old infant: a case report

**DOI:** 10.1186/s13019-017-0612-6

**Published:** 2017-06-12

**Authors:** Fuyang Mei, Bing Zhou, Yong Cui

**Affiliations:** Department of Cardiothoracic Surgery, Zhejiang Provincial People’s Hospital, No. 158 Shangtang Road, Hangzhou, Zhejiang 310014 People’s Republic of China

**Keywords:** Cardiac tumor, Fibroma, Right ventricular outflow tract obstruction, Surgery

## Abstract

**Background:**

Cardiac fibroma is rarely encountered in children, and even more rare in neonates. We herein report a case of a 5-month-old female with severe right ventricular outflow tract obstruction caused by a large right ventricle fibroma that was successfully surgically resected.

**Case presentation:**

This report describes the case of a 5-month-old female infant with a large mass measuring 26 × 22 mm in the right ventricle cured successfully with surgery. Physical examination revealed a harsh S1 sound and a grade IV systolic murmur on the left sternal border. Surgical resection was indicated due to severe right ventricular outflow tract obstruction and further follow-up evaluation was uneventful.

**Conclusion:**

The surgical procedure to excise such a large cardiac fibroma in a 5-month-old infant is feasible and safe.

## Background

Cardiac fibroma is rarely encountered in children, and even more rare in neonates [1–4]. We herein report a case of a 5-month-old female with severe right ventricular outflow tract obstruction caused by a large right ventricle fibroma that was successfully surgically resected.

## Case presentation

**Fig. 1:**
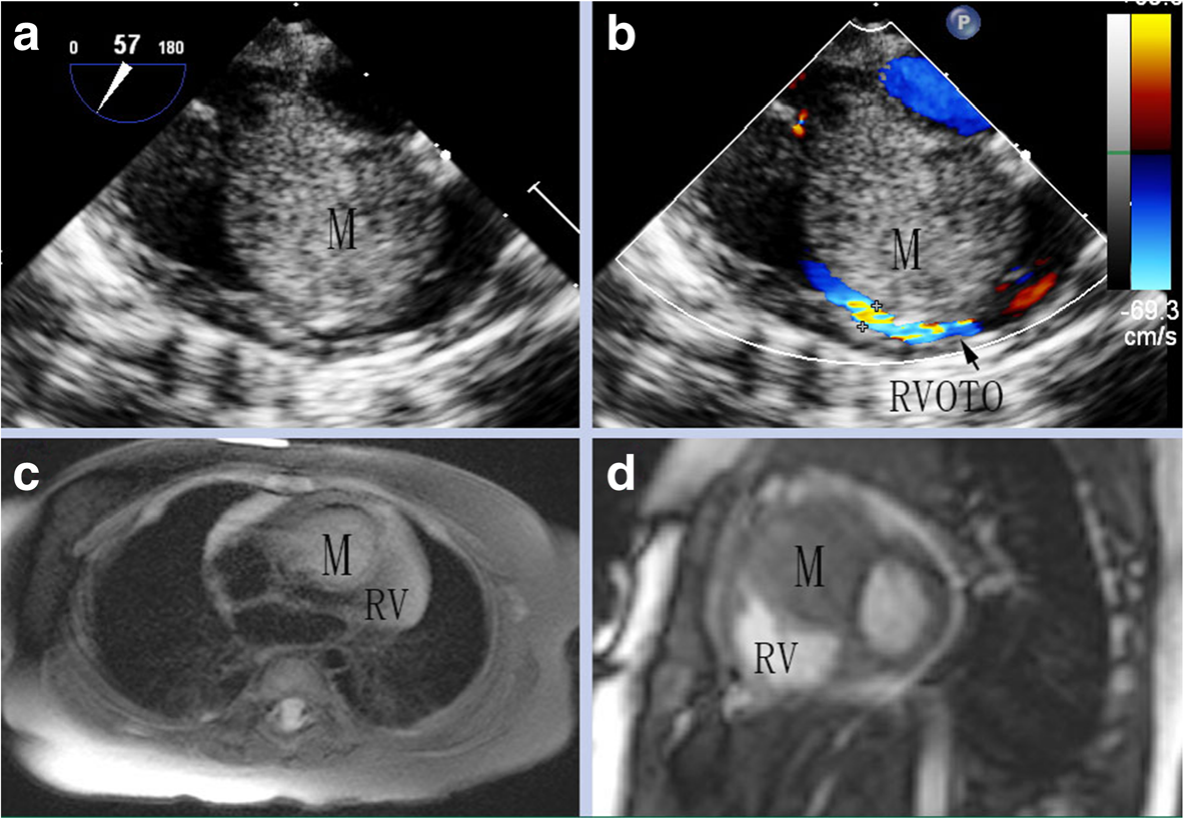
Image information demonstration the mass in the RV. **a** Transesophageal echocardiography. **b** Right ventricular outflow tract obstruction. **c**, **d** The Cardiac magnetic resonance imaging. M indicates mass, RV indicates right ventricle, RVOTO indicates right ventricular outflow tract obstruction

A 5-month-old female presented to our hospital with a harsh S1 sound and a grade IV systolic murmur on the left sternal boarder. A transthoracic echocardiogram was performed, which revealed a homogeneous mass measuring 26 × 22 mm (Fig. 1a), with a broad base from ventricular septum and the body protruding into the right ventricular chamber, causing an accelerated right ventricular outflow (Vmax = 4.0 m/s, PG = 64 mmHg) with an obstruction (Fig. 1b). At the time of admission, the overall pediatric evaluation was normal including the laboratory data, chest radiograph and electrocardiogram. The cardiac-magnetic resonance imaging confirmed the presence of a solid mass appending to the right side of ventricular septum (Fig. 1c-d). However, all of the radiological examinations did not reveal the pathological nature of the mass.

**Fig. 2:**
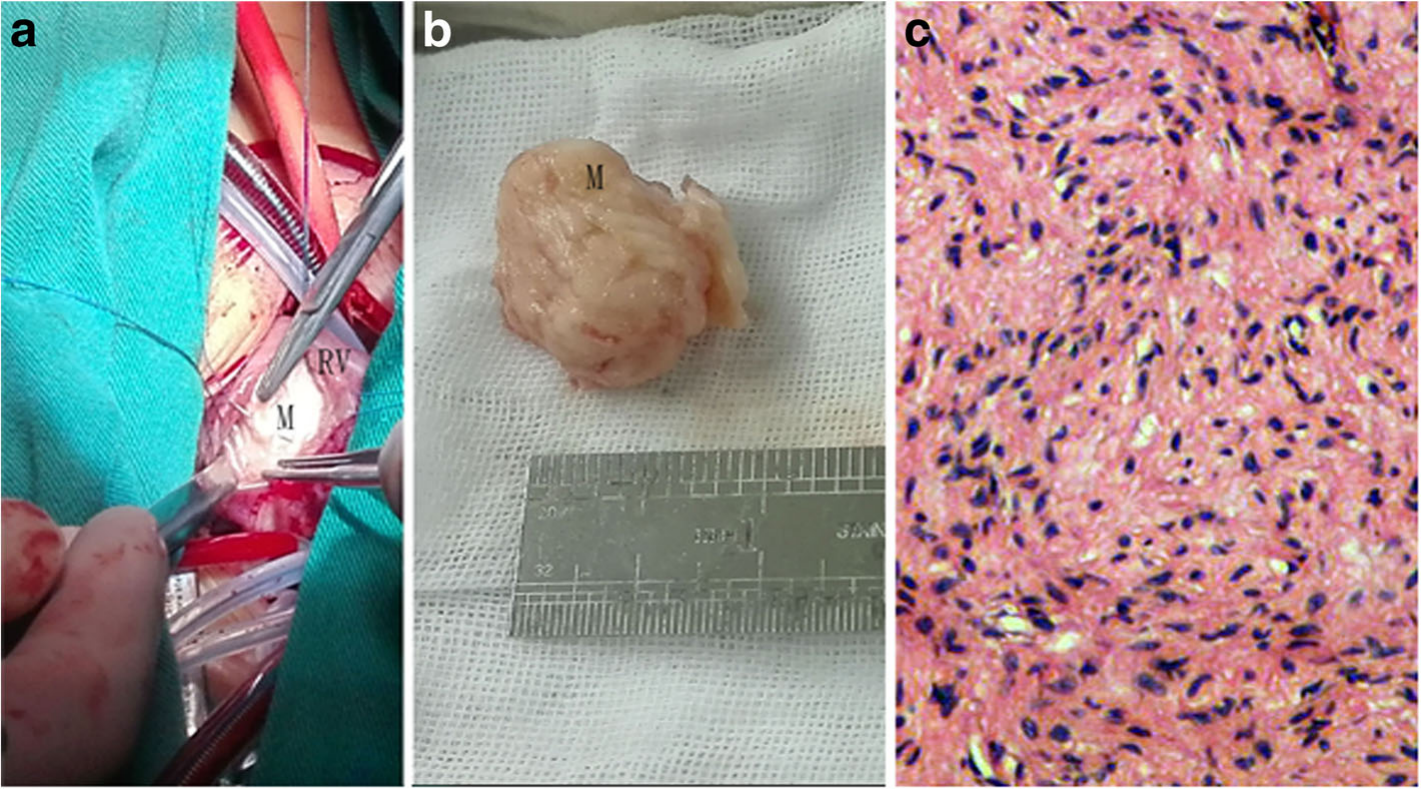
Surgical view and Microscopic photograph. **a** The appearance of the tumor after incision of RV; **b** The mass was resected and measured. **c** Microscopic photograph of the removed mass revealing fibroma consist of spindle fibroblasts with collagen deposition. M indicates mass, RV, right ventricle

Considering the size and location of the mass as well as the potential complications, surgery was the best option for this patient. As soon as the pericardium was incised after median sternotomy, a tenacious mass was palpable on the right ventricular free wall. Under cardiopulmonary bypass, an incision was made on the free wall of right ventricle, exposing a white, tenacious, non-capsulated egg-like neoplastic growth protruding into the right ventricular chamber, with its base arising from interventricular septum. The body of the tumor was adjacent to the right ventricular outflow tract, causing an obstruction (Fig. 2a). The tumor was resected along with its base, measuring 27 × 25 × 22 mm (Fig. 2b), and the incision on the right ventricle was primarily closed. The intra-operative pathological screening reported that the specimen was consisted with fibroblasts along with collagen deposition, combined with the negative results reported from the microbiology, which confirmed the diagnosis of cardiac fibroma (Fig. 2c). The patient’s postoperative course was uneventful and got discharged by the expected duration. There was neither any recurrence of the tumor nor any complications during the 3-month follow-up evaluation with a normal cardiac function.

## Discussion and conclusion

Among congenital heart tumors reported, there are mainly rhabdomyomas and less frequently fibromas. The incidence of primary cardiac tumors in children has been reported to be 0.03–0.32%, with fibromas accounting for 25%. Approximately 70% of primary cardiac tumors are benign with a morbidity rate of around 0.27 to 0.8% [1–4]. Cardiac fibromas normally develop from the residing fibroblasts or myofibroblasts of the heart. Cardiac Fibromas are the most common condition to cause clinically significant arrhythmias even though they are pathologically benign. The presenting symptoms include arrhythmias (32%), murmurs (20%) and abnormal chest radiographs (20%) depending on the size and location of the tumor [5]. The diagnosis of cardiac fibroma does not solely depends on physical examination, X-ray, echocardiogram, computed tomography and cardiac-magnetic resonance imaging. Biopsy should also be done for confirmation [5, 6]. However, the gradually growing fibromas make it difficult to diagnose during the early stages of the condition, as the patient would remain asymptomatic, until later, the patient may present with chest pains, conduction abnormalities and ventricular inflow/outflow tract obstructions that may affect the quality of life.

Management strategy of fibromas remain controversial. To avoid the complications, such as heart inflow/outflow tract obstructions and malignant arrhythmias, some may think that early surgical intervention is the first choice of treatment when cardiac tumors, probably fibromas, are diagnosed. If the excision of the fibroma affects the integrity of the ventricle and the arrhythmias can be managed with pharmacological agents, the surgery can be postponed until the inflow/outflow tract obstruction appears [6–8].

As the fibromas are most commonly found in the left ventricular free wall or septum, the reporting case where the fibroma occupies most of the right ventricular chamber, is a comparatively rare form. In this case, the tumor reached a dimension of 26 × 22 mm and protruded to the right ventricle causing an obvious obstruction. Surgical removal of the tumor was performed soon after admission, as there is a major risk for sudden death due to conductive tissue involvement.

Till now, right ventricular outflow tract obstruction caused by such a massive fibroma in a very young patient has never been reported. Considering that fibromas are much likely to increase in size, early resection is recommended in such patients for a successful surgical outcome and at the same time avoiding potential abnormalities and cardiovascular collapse in the later life. In addition, our experience showed that the surgical procedure to excise such a large cardiac fibroma in a 5-month-old infant is feasible and safe.
